# The clinical impact of serum 1,5-anhydro-D-glucitol levels on coronary artery calcification and adverse outcomes assessed by coronary optical coherence tomography in diabetic patients

**DOI:** 10.3389/fcvm.2022.997649

**Published:** 2022-08-30

**Authors:** Hsin-I Teng, Hsiang-Yao Chen, Chuan-Tsai Tsai, Wei-Chieh Huang, Ying-Ying Chen, Chien-Hung Hsueh, William K. Hau, Tse-Min Lu

**Affiliations:** ^1^Heart Center, Cheng Hsin General Hospital, Taipei, Taiwan; ^2^Department of Internal Medicine, School of Medicine, College of Medicine, National Yang Ming Chiao Tung University, Taipei, Taiwan; ^3^TaiVeCoron Study Group, Taipei Veterans General Hospital Coronary Intervention Study Group, Taipei, Taiwan; ^4^Department of Internal Medicine, Taipei Hospital, Ministry of Health and Welfare, Taipei, Taiwan; ^5^Division of Cardiology, Department of Internal Medicine, Taipei Veterans General Hospital, Taipei, Taiwan; ^6^Division of Nephrology, Department of Internal Medicine, MacKay Memorial Hospital, Taipei, Taiwan; ^7^Department of Medicine and Therapeutics, The Chinese University of Hong Kong, Shatin, Hong Kong SAR, China; ^8^Department of Health Care Center, Taipei Veterans General Hospital, Taipei, Taiwan

**Keywords:** 1, 5-anhydro-D-glucitol, coronary artery calcification, optical coherence tomography, diabetes mellitus, 1,5-AG

## Abstract

**Background:**

Serum 1,5-anhydro-D-glucitol (1,5-AG) is a novel biomarker for short-term glycemic status and postprandial hyperglycemia. The association between serum 1,5-AG levels and coronary artery calcification (CAC) through a quantitative assessment using optical coherence tomography (OCT) is unclear. We aimed to evaluate this association using OCT in patients with diabetes mellitus (DM).

**Methods:**

From June 2016 to December 2019, we prospectively enrolled 256 patients who underwent OCT-guided percutaneous coronary intervention (PCI). Half of the patients had diabetes. Patients were followed up for a mean period of 1.8 ± 0.8 years (median: 2.2 years). The relative calcium index and relative lipid core index measured by quantitative OCT analysis were used to evaluate the intra-plaque calcium and lipid levels of culprit plaques. We also analyzed the correlation between serum 1,5-AG levels and long-term major adverse cardiovascular events.

**Results:**

Serum 1,5-AG levels were significantly lower in diabetic patients than in non-diabetic patients (DM vs. non-DM: 55.6 ± 27.9 μg/mL vs. 63.7 ± 26.1 μg/mL, *p* = 0.016), and lower in fibrocalcified lesions than in fibrotic or fibrolipidic lesions (fibrocalcified vs. fibrotic or fibrolipidic: 42.8 ± 19.1 vs. 72.9 ± 25.2 or 66.4 ± 27.5 μg/mL, *p* < 0.001, respectively). In addition, we found a significant inverse correlation between serum 1,5-AG levels and relative calcium index (*r* = −0.729, *p* < 0.001). In multivariate Cox regression analysis, low serum 1,5-AG level was identified as an independent predictor for major adverse cardiovascular events in diabetic patients (*p* = 0.043), but not in non-diabetic patients (*p* = 0.748) after adjusting for age and sex.

**Conclusion:**

This study revealed that low serum 1,5-AG levels were associated with an increased risk of CAC as assessed by OCT, especially in diabetic patients. Low serum 1,5-AG levels may predict future major adverse cardiovascular events in diabetic patients undergoing OCT-guided PCI.

## Introduction

Coronary artery calcification (CAC), an important marker of coronary artery disease (CAD), is known to correlate with advanced atherosclerosis (1, 2), and cardiovascular outcomes in CAD patients with or without percutaneous coronary intervention (PCI) (3–6). Among patients with diabetes mellitus (DM), CAC tends to be higher and serves as an independent risk factor for subsequent major adverse cardiac events (MACE) (7). In contrast, hyperglycemia may lead to a pre-atherogenic effect, resulting in the development of calcified coronary plaques. Moreover, elevated glycosylated hemoglobin (HbA1c) independently contributes to severe CAC and its progression, suggesting that poor glycemic control may play an essential role in CAC pathophysiology (8). Notably, serum 1,5-anhydro-D-glucitol (1,5-AG) is a novel biomarker for short-term glycemic status and postprandial hyperglycemia, which may be more sensitive than HbA1c as a marker of postprandial glycemic control (9, 10). In patients undergoing elective PCI, low pre-procedural and follow-up serum 1,5-AG levels have been reported to be associated with MACE, even in patients with HbA1c < 7.0% (11, 12). All current evidence suggests that serum 1,5-AG levels may be a novel risk factor for coronary atherosclerosis.

Several studies on intravascular imaging, including intravascular ultrasound (IVUS) and optical coherence tomography (OCT), have shown a close relationship between CAC and the extent of atherosclerotic plaques (2, 13, 14). Recently, an IVUS study reported that a low 1,5-AG level, indicating postprandial hyperglycemia, is associated with the severity of CAC (15). However, calcium obstructs ultrasound penetration, meaning IVUS is unable to measure calcium thickness, area, or volume (16, 17). In contrast, OCT has an extraordinarily higher resolution than IVUS (10–20 vs. 100–200 μm, respectively) and has been suggested as a preferred imaging modality for the detailed *in vivo* assessment of atherosclerotic plaques. It can provide detailed information on plaque morphology and tissue composition, including intra-plaque lipidic arc and calcium thickness, area, volume (18). Therefore, in this study, we aimed to evaluate the relationship between serum 1,5-AG levels and CAC assessed by OCT in diabetic patients.

## Materials and methods

### Study design and objectives

**FIGURE 1:**
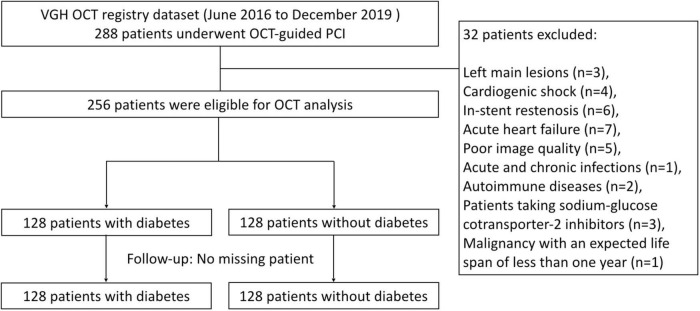
Study flow.

From June 2016 to December 2019, 288 patients with symptomatic angiographically significant stenotic coronary lesions (defined as lumen diameter stenosis ≥ 70%) were enrolled in the study. Patients consented to the use of their medical information prior to investigation. We selected the most severe lesion as the target lesion for OCT analysis in patients with multiple vessel diseases. Of enrolled patients, 256 patients were eligible for OCT analysis. The other 32 patients were excluded according to the exclusion criteria. The exclusion criteria for this study were patients with in-stent restenosis, left main lesions, cardiogenic shock, acute decompensated congestive heart failure, poor image quality, acute and chronic infections, autoimmune diseases, and malignancy with an expected life span of less than 1 year. Patients taking sodium-glucose cotransporter 2 inhibitors were also excluded to reduce possible interference, avoiding falsely low serum 1,5-AG levels (19, 20) (Figure 1). This specific research question was developed *post hoc*. However, data were collected prospectively for the purpose of analyses such as this. DM was defined according to the American Diabetes Association criteria or medical records and the treatment with insulin or glucose-lowering medication. Hypertension was diagnosed as systolic blood pressure ≥ 140 mmHg or diastolic blood pressure ≥ 90 mmHg or the use of oral antihypertensive medicines. Hyperlipidemia was defined according to the modified National Cholesterol Education Program-Adult Treatment Panel III. According to 2014 ACC/AHA guideline for Non–ST-Elevation Acute Coronary Syndromes (ACS) and 2017 Guidelines on Management of Acute Myocardial Infarction in Patients Presenting with ST-Segment Elevation, ACS was defined as a composite of unstable angina pectoris, non-ST-segment elevation myocardial infarction (NSTEMI) and ST-segment elevation myocardial infarction (STEMI). The estimated glomerular filtration rate (eGFR) was calculated using the Modification of Diet in Renal Disease (MDRD) formula (21). The study conformed to the principles outlined in the Declaration of Helsinki and was approved by relevant ethical bodies. The Institutional Review Board approved the study protocol at the Taipei-Veterans General Hospital, and all participants gave their written informed consent for their data to be used for research.

### Procedural details and angiographic analysis

During PCI procedures, selecting the imaging modality or not was dependent on the operator’s preference and clinical scenario. Only if OCT was chosen as the imaging modality would patients be enrolled. All enrolled patients underwent OCT imaging prior to PCI. If the OCT catheter could not cross the target lesion, predilation with a 1.5 mm or 2.0 mm balloon would be performed. A total of 256 patients were eligible for OCT analysis, and all of them underwent successful OCT-guided PCI. OCT-guided PCI was considered successful if the stent optimization criteria were met: (1) minimal stent area of ≥ 4.5 mm^2^; (2) minimal stent area of ≥ 80% of the average reference lumen area; (3) stent-adjacent vessel lumen distance of ≤ 200 μm; (4) edge dissection with a width of < 200 μm adjacent (<5 mm) to a stent edge; (5) intrastent plaque/thrombus protrusion of < 500 μm in thickness; and (6) reference luminal narrowing of ≥ 4.5 mm^2^. Coronary thrombolysis in myocardial infarction (TIMI) grade 3 flow was obtained at the end of the procedure without major complications. Dual antiplatelet therapy was started after the procedure, and all patients received aspirin (100 mg per day) indefinitely and clopidogrel (300 mg loading dose, and 75 mg maintenance per day) for 12 months. Medication for the treatment of angina pectoris (calcium channel blockers, beta-blockers, and nitrates) were also continued. The operation records and medical records were thoroughly reviewed, and all demographic and procedural variables were recorded in the data collection sheet. The following angiographic parameters were obtained by quantitative coronary analysis (QCA): minimal lumen diameter (MLD, mm), reference vessel diameter (RVD, mm), and percentage diameter stenosis (% DS).

### Optical coherence tomography image analysis

The target plaque lesion was evaluated by pre-intervention OCT in all enrolled patients. By comparing pre- and post-PCI OCT findings, a target lesion segment was defined as the lesion that was stented. We performed OCT pullbacks with the ILUMIEN OPTIS™ imaging system and Dragonfly™ (Abbott Vascular, Santa Clare, CA, United States) OCT catheters. Two independent investigators (WC Huang and HY Chen) analyzed the OCT images using the offline ORW software (Abbott Vascular, Santa Clare, CA, United States). If the results differed between the two investigators, a third independent investigator was consulted to act as an umpire. OCT imaging was assessed at 0.1-mm intervals, and longitudinal cross-sections were analyzed within the stented lesion, 5 mm proximally and distally to the stent edge.

According to the OCT analysis, atherosclerotic plaques were classified as fibrotic, fibrolipidic, or fibrocalcified (22). The detailed OCT definitions were as follows: (1) fibrotic type, maximum lipid core arc ≤ 90^°^ and maximum calcium arc ≤ 90^°^; (2) fibrolipidic type, which is sub-classified as fibroatheroma, maximum lipid core arc > 90^°^, minimal fibrous cap thickness > 65 μm, and thin-cap fibroatheroma (TCFA), maximum lipid core arc > 90^°^ and minimal fibrous cap thickness ≤ 65 μm at the thinnest part; and (3) fibrocalcific type, maximum calcium arc > 90^°^ with maximum lipid core arc ≤ 90^°^. Quantitative analysis of the calcium content in native vessels was performed using cross-sectional OCT images at 0.1-mm intervals. The minimal lumen area, maximal calcium arc, and thickness of the calcification were recorded. We measured the relative calcium index [RCI = (mean calcium arc × calcium length)/(360 × analyzed length)] and relative lipid core index [RLCI = (mean lipid core arc × lipid core length)/(360 × analyzed length)] as the relative volumetric indices of intra-plaque calcium and lipid content. A calcium nodule was defined as an accumulation of multiple small protruding nodular calcifications with a superficial thrombus or fibrin.

### Laboratory measurements

Blood samples were collected prior to the PCI procedure and immediately centrifuged at 3,000 rpm for 10 min at 4°C. Plasma samples were kept frozen at −80°C until analysis. Serum 1,5-AG was measured using a commercial competitive ELISA kit (Cloud-Clone Corp., Houston, United States) with a standard range of 1.9–150 μg/mL. The detection limit was 0.7 μg/mL.

### Clinical follow-up and outcomes

All patients were followed up by medical records review and telephone interviews. MACE was defined as the composite endpoint consisting of cardiovascular (CV) death, non-fatal myocardial infarction (MI), target vessel revascularization (TVR), and non-target vessel revascularization (Non-TVR). CV death was diagnosed as any death with a definite CV cause or death that is not clearly attributed to a non-CV cause. Non-fatal myocardial infarction was defined as the presence of significant new Q waves in at least two electrocardiography leads or symptoms compatible with MI associated with an increase in creatinine kinase-MB fraction ≥ 3 times the upper limit of the reference range. TVR referred to clinically driven repeat revascularization in the follow-up period due to restenosis within the target lesion or the same epicardial coronary artery. No patients were lost to follow-up.

### Statistical analysis

All continuous variables are presented as mean ± standard deviation (SD) or 95% confidence interval (CI) and median [interquartile range (IQR)]. Categorical variables are described as frequencies. The unpaired *t*-test or Mann–Whitney *U*-test were used for comparing continuous variables, as appropriate. Pearson’s correlation coefficients were calculated to examine the possible correlations between continuous variables. Comparison of serum 1,5-AG levels among plaque types was performed by analysis of variance (ANOVA). *Post hoc* comparisons were performed by the Bonferroni test. Univariate and multivariate Cox regression models were used to examine the association between clinical outcomes and OCT plaque characteristics. Hazard ratios (HRs) and 95% CIs were calculated. Statistical significance was defined at *p* < 0.05. All statistical analyses were performed using the SPSS statistical software (IBM SPSS Statistics for Windows, Version 22.0. Armonk, NY: IBM Corp.).

## Results

### Baseline characteristics of the study population

**FIGURE 2:**
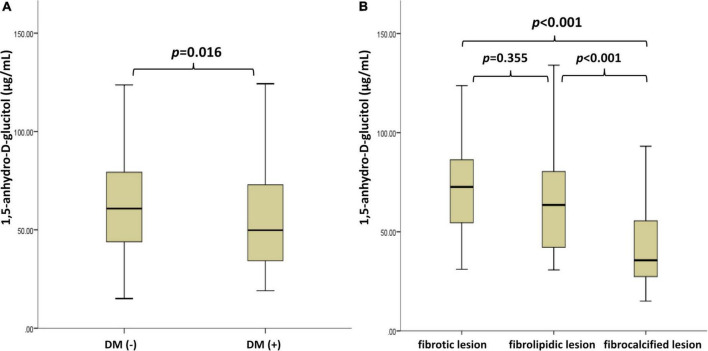
Serum 1,5-AG levels were stratified by DM and plaque characteristics. *p*-values by analysis of variance (ANOVA and Chi-square) were shown. *Post hoc* comparisons were performed by the Bonferroni test. **(A)** Serum 1,5-AG level was significantly lower in DM patients than in non-DM patients (DM vs. non-DM: 55.6 ± 27.9 μg/mL vs. 63.7 ± 26.1 μg/mL, *p* = 0.016). **(B)** Serum 1,5-AG levels were significantly lower in fibrocalcified lesions than in fibrotic or fibrolipidic lesions (fibrocalcified vs. fibrotic or fibrolipidic: 42.8 ± 19.1 vs. 72.9 ± 25.2 or 66.4 ± 27.5 μg/mL, *p* < 0.001). 1,5-AG, 1,5-anhydro-D-glucitol, DM, diabetes mellitus.

From June 2016 to December 2019, 288 patients with symptomatic angiographically significant stenotic coronary lesions who underwent OCT-guided PCI were enrolled in the study. Of them, 256 patients were eligible for OCT analysis. 32 patients were excluded according to the exclusion criteria. Baseline characteristics of the 256 patients stratified by DM are summarized in Table 1. The mean age of the patients was 66 ± 12 years. Half of the patients had DM (50%, 128/256), and the majority of patients were male (79%, 201/256). The mean serum 1,5-AG level of the total population was 59.6 ± 27.2 μg/mL. Moreover, serum 1,5-AG level was significantly lower in DM patients than in non-DM patients (DM vs. non-DM: 55.6 ± 27.9 μg/mL vs. 63.7 ± 26.1 μg/mL, *p* = 0.016, Figure 2A).

**TABLE 1:** Baseline characteristics of enrolled patients stratified by DM.

Variables	Overall patient (*N* = 256)	Non-DM patients (*N* = 128)	DM patients (*N* = 128)	*p*-value
**Baseline characteristics, *n* (%)**				
Age (year)	66 ± 12	65 ± 13	68 ± 12	0.074
Male	201 (79)	112 (88)	89 (70)	< 0.001
Hypertension	193 (75)	91 (71)	102 (80)	0.110
Atrial fibrillation	22 (9)	12 (9)	10 (8)	0.656
Hypercholesterolemia	181 (71)	88 (69)	93 (73)	0.492
Prior MI	42 (16)	21 (16)	21 (16)	0.999
Prior CVA	25 (10)	10 (8)	15 (12)	0.292
PAOD	12(5)	3 (2)	9 (7)	0.076
**Clinical presentation, *n* (%)**				0.822
Stable angina	196 (77)	96 (75)	100 (78)	
NSTEMI	45 (17)	25 (19)	20 (16)	
STEMI	15 (6)	7 (6)	8 (6)	
**Medications, *n* (%)**				
Dual antiplatelet therapy	212 (83)	108 (84)	104 (81)	0.886
Statin	158 (62)	76 (59)	82 (64)	0.393
Beta-blocker	115 (45)	55 (43)	60 (47)	0.493
ACEI/ARB	122 (48)	60 (47)	62 (48)	0.884
**Laboratory data**				
T. Cholesterol (mg/dL)	157 ± 41	161 ± 43	151 ± 38	0.064
LDL-C (mg/dL)	92 ± 35	97 ± 37	87 ± 32	0.019
eGFR	61 ± 28	62 ± 24	58 ± 27	0.768
FBS	123 ± 54	99 ± 23	147 ± 64	< 0.001
HbA1c	7.1 ± 1.4	5.8 ± 0.5	7.5 ± 1.4	< 0.001
1,5-AG (μg/mL)	59.6 ± 27.2	63.7 ± 26.1	55.6 ± 27.9	0.016

1,5-AG, 1,5-anhydro-D-glucitol; ACEI/ARB, angiotensin-converting enzyme inhibitors/angiotensin II receptor blockers; CVA, cerebrovascular accident; DM, diabetes mellitus; eGFR, estimated glomerular filtration rate, FBS, fasting blood sugar; HbA1c, Hemoglobin A1c; LDL-C, Low-density lipoprotein cholesterol; MI, myocardial infarction; MLD, minimal lumen diameter; NSTEMI, non ST-segment elevation myocardial infarction; PAOD, peripheral arterial occlusive disease; STEMI, ST-segment elevation myocardial infarction.

OCT characteristics of the lesions are summarized in Table 2. According to the OCT analysis, the evaluated atherosclerotic lesions were classified as fibrotic plaque (41.8%, 107/256), fibrolipidic plaque (20.0%, 46/256), and fibrocalcific plaque (40.2%, 103/256). TCFAs were present in 13 patients (5.1%), while calcium nodules were present in 22 patients (8.6%). Complex lesions, defined as fibrolipidic and fibrocalcified lesions, were more common among patients with DM. In addition, calcium and lipid contents (RCI = 0.18 ± 0.12, RLCI = 0.065 ± 0.067, *p* < 0.001, respectively), TCFA (8.6%, *p* = 0.01), calcium arc (98.2 ± 42.1°, *p* < 0.001), and calcium length (13.2 ± 5.62 mm, *p* < 0.001) were higher in DM patients.

**TABLE 2:** Angiographic characteristics and OCT assessments of enrolled patients stratified by DM.

Variables	Overall patient (*N* = 256)	Non-DM patients (*N* = 128)	DM patients (*N* = 128)	*p*-value
**Angiographic characteristics**				
**Stent type, n (%)**				
Bare-metal stent	46 (18)	26 (20)	20 (16)	0.881
Drug-eluting stent (second-generation)	210 (82)	102 (80)	108 (84)	0.921
Duration of stent implantation (months)	24 ± 12	23 ± 11	26 ± 13	0.877
**Diseased vessel number**				0.922
Single-vessel disease	102 (40)	54 (42)	48 (38)	
Double-vessel disease	79 (31)	38 (30)	41 (32)	
Triple-vessel disease	75 (29)	36 (28)	39 (31)	
**QCA measurements**				
Minimal lumen diameter (mm)	1.4 ± 0.5	1.4 ± 0.5	1.4 ± 0.5	0.892
Reference vessel diameter (mm)	2.6 ± 0.5	2.7 ± 0.6	2.6 ± 0.5	0.632
Diameter stenosis (%)	79 ± 16	81 ± 17	78 ± 15	0.843
**OCT assessments**				
**Qualitative assessments, n (%)**				<0.001
Fibrotic lesion	107 (42)	84 (66)	23 (18)	<0.001
Fibrolipidic lesion	46 (20)	12 (9.4)	34 (26)	<0.001
Fibrocalcified lesion	103 (40)	32 (25)	71 (56)	<0.001
Thin-cap fibroatheroma	13 (5.1)	2 (1.6)	11 (8.6)	0.010
Calcium nodule	22 (8.6)	10 (7.8)	12 (9.3)	0.669
**Quantitative parameters**				
Minimal lumen area (mm^2^)	2.2 [1.7, 2.8]	2.2 [1.7, 2.7]	2.2 [1.6, 2.8]	0.844
Minimal lumen diameter (mm)	1.1 ± 0.5	1.1 ± 0.5	1.1 ± 0.5	0.833
Relative calcium index	0.10 [0.06, 0.28]	0.09 [0.03, 0.12]	0.19 [0.07, 0.29]	<0.001
Relative lipid core index	0.03 [0.02, 0.04]	0.03 [0.02, 0.04]	0.07 [0.02, 0.11]	<0.001
Mean max calcium arc (degree)	82 [38, 162]	46 [23, 92]	106 [54, 202]	<0.001
Mean max calcium length (mm)	11.2 ± 5.1	8.2 ± 4.6	13.2 ± 5.6	<0.001
Mean max calcium thickness (mm)	0.76 ± 0.24	0.66 ± 0.21	0.86 ± 0.25	0.342
Mean max lipid core arc (degree)	32 [13, 64]	21 [8, 42]	45 [25, 89]	<0.001
Mean max lipid core length (mm)	4.3 ± 1.2	3.3 ± 1.3	5.8 ± 1.6	0.026

Data are given as number (percentage) for categorical variables and mean ± standard deviation or median (IQR) for continuous variables. DM, diabetes mellitus; OCT, optical coherence tomography; QCA, qualitative comparative analysis.

**FIGURE 3:**
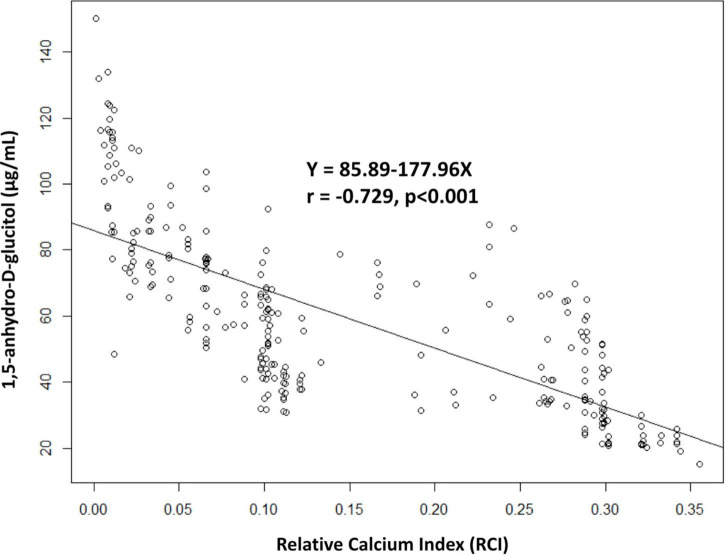
Correlations between serum 1,5-AG level and relative calcium index. 1,5-AG, 1,5-anhydro-D-glucitol.

**FIGURE 4:**
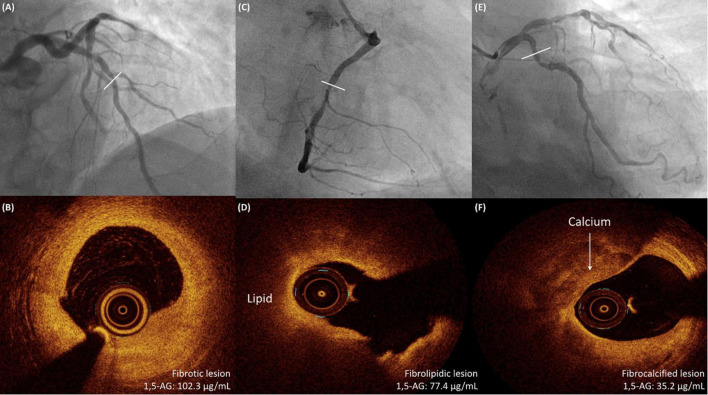
Three representative cases with different correlations between OCT-detected plaque type and serum 1,5-AG levels. **(A,B)** A 68-year-old man with hypertension and stable angina is shown to have a lesion in the middle LAD on angiography. OCT examination shows a fibrotic lesion. The serum 1,5-AG level is 102.3 μg/mL. **(C,D)** A 72-year-old man with DM and NSTEMI is shown to have a severe lesion in the middle RCA on angiography. OCT examination shows a fibrolipidic lesion. The serum 1,5-AG level is 77.4 μg/mL. **(E,F)** A 68-year-old man with DM and stable angina is shown to have a diffuse lesion in the proximal LCX on angiography. OCT examination shows a fibrocalcified lesion. The serum 1,5-AG level is 35.2 μg/mL. 1,5-AG, 1,5-anhydro-D-glucitol; DM, diabetes mellitus; LAD, left anterior descending artery; LCX, left circumflex artery; NSTEMI, non ST-segment elevation myocardial infarction; OCT, optical coherence tomography; RCA, right coronary artery.

Levels of serum 1,5-AG were significantly lower in fibrocalcified lesions than in fibrotic or fibrolipidic lesions (fibrocalcified vs. fibrotic or fibrolipidic: 42.8 ± 19.1 vs. 72.9 ± 25.2 or 66.4 ± 27.5 μg/mL, *p* < 0.001, respectively, Figure 2B). Furthermore, we found a strong inverse correlation between serum 1,5-AG level and the RCI (*r* = −0.729, *p* < 0.001, Figure 3). In contrast, serum 1,5-AG levels correlated significantly to the RLCI in ACS patients (*r* = −0.358, *p* < 0.001), but not in non-ACS patients (*r* = −0.062, *p* = 0.322). Three representative cases with different correlations between plaque type and serum 1,5-AG levels are shown in Figure 4.

All patients were followed up completely for a mean period of 1.8 ± 0.8 years (median: 2.2 years, IQR: 1.0–2.5 years). During the follow-up period, 49 patients experienced MACE (19.1%), including 4 CV deaths (1.6%), 7 non-fatal MIs (2.7%), 30 TVRs (11.7%), and 8 Non-TVRs (3.1%) (Table 3). A significantly lower 1,5-AG level was found in patients who experienced MACE compared to those who did not (47.3 vs. 61.9 μg/mL, *p* = 0.006). The best cut-off value of serum 1,5-AG for discrimination between patients with and without MACE was 34.67 μg/mL (area under the curve = 0.636). In the multivariate Cox regression analysis, low serum 1,5-AG levels remained an independent predictor of MACE in DM patients (*p* = 0.043, Table 4), but not in non-DM patients (*p* = 0.748) after adjusting for age and sex.

**TABLE 3:** Clinical outcomes of enrolled patients stratified by DM.

Variables	Overall patient (*N* = 256)	Non-DM patients (*N* = 128)	DM patients (*N* = 128)	*p*-value
**Outcomes, *n (%)***				
MACE	49 (19)	10 (8)	39 (30)	<0.001
CV death	4 (2)	0 (0)	4 (3)	0.044
Non-fatal MI	7 (3)	1 (1)	6 (5)	0.055
TVR	30 (12)	6 (5)	24 (19)	<0.001
Non-TVR	8 (3)	3 (2)	5 (4)	0.621

DM, diabetes mellitus; CV, cardiovascular; MACE, major adverse cardiac events; MI, myocardial infarction; TVR, target vascular revascularization.

**TABLE 4:** Cox regression analysis for long-term MACE in enrolled patients stratified by DM.

All patients	Univariate		Multivariate	
	HR (95% CI)	*p-*value	HR (95% CI)	*p*-value
Age	1.002 (0.970–1.028)	0.821	1.001 (0.968–1.030)	0.882
Sex	2.615 (1.290–5.251)	0.007	2.081 (1.018–4.251)	0.042
DM	4.733 (1.961–11.505)	0.001	3.721 (1.501–9.227)	0.003
1,5-AG	0.981 (0.961–0.990)	0.021	0.983 (0.971–1.000)	0.054
**DM patients**				
Age	0.997 (0.962–1.038)	0.832	1.006 (0.970–1.031)	0.741
Sex	2.415 (1.130–5.130)	0.025	2.048 (1.210–5.602)	0.011
1,5-AG	0.986 (0.960–1.002)	0.061	0.980 (0.961–1.001)	0.043
**Non-DM patients**				
Age	1.002 (0.937–1.059)	0.964	1.001 (0.938–1.071)	0.983
Sex	0.000 (0.000—-)	0.999	0.000 (0.000—-)	0.999
1,5-AG	0.991 (0.958–1.029)	0.706	0.993 (0.962–1.031)	0.748

1,5-AG, 1,5-anhydro-D-glucitol; CI, confidence intervals; DM, diabetes mellitus; HR, hazard ratio; MACE, major adverse cardiovascular event.

## Discussion

Our study demonstrated that low serum 1,5-AG is associated with an increased risk of CAC, as assessed by OCT, especially in patients with DM. Moreover, serum 1,5-AG levels may predict future MACE in patients with DM undergoing OCT-guided PCI. To the best of our knowledge, this study is the first to investigate the correlation between serum 1,5-AG and CAC in DM patients using OCT analysis.

Increasing evidence has shown that postprandial hyperglycemia and glucose fluctuations affect coronary atherosclerosis progression (23–25). 1,5-AG is a natural 1-deoxy form of glucose in the diet. Most 1,5-AG, filtered in the glomerulus, is reabsorbed in the renal tubules, which is competitively inhibited by glucose. Therefore, as serum glucose levels exceed the renal threshold for glucosuria, the resorption of 1,5-AG is suppressed, leading to a rapid reduction in serum 1,5-AG (26). Recent studies have suggested that serum 1,5-AG concentration may be a useful clinical marker of postprandial hyperglycemia and glycemic excursions (9, 27–29), and therefore a better glycemic biomarker than HbA1c in reflecting glycemic excursions (9). Growing *in vitro* and *in vivo* evidence has linked postprandial glucose (PPG) excursions to diabetic calcification, likely through the mechanisms of oxidative stress generation, inflammatory mediator activation, and endothelial dysfunction (30, 31). Therefore, we hypothesized that 1,5-AG may be associated with coronary calcification. Wada et al. demonstrated that low serum 1,5-AG levels were a significant predictor of a greater calcification angle assessed by IVUS (15). However, volumetric indices based on calcium length were not available in Wada’s IVUS study. Compared with IVUS, OCT can assess calcium length, thickness, area, and volumetric indices of calcium content (RCI) (22, 32–34). This is because, compared to IVUS, OCT has a higher resolution and can also penetrate calcified deposits (32). Moreover, OCT has exhibited even higher accuracy for diagnosing calcium with excellent sensitivity and specificity in a histological validation study (35). Taking advantage of the superiority of OCT in image analysis, our data showed that low serum 1,5-AG levels were associated with fibrocalcified lesions and strongly inversely correlated with the RCI, especially in DM patients.

CAC tended to be higher in diabetic patients (7), which was concomitant with the increase in total plaque volume (36, 37) and represented an independent risk factor for adverse cardiovascular events (3–6), even independent of diabetic status (7). In addition, the prevalence of TCFA seems to be higher in both culprit and non-culprit lesions among diabetic patients with or without ACS at presentation and may lead to a higher rate of MACE (38–43). Our study showed consistently a higher percentage of CAC and TCFA in DM group. However, the management of calcified vessels remains clinically challenging. Severe CAC may increase complication rates of interventional procedures, and patients with heavily calcified lesions have been shown to have a poor prognosis (4, 44). On the other hand, accumulating evidence has reported the prognostic value of 1,5-AG for long-term complications in patients with DM. Low serum 1,5-AG levels may identify patients at high cardiovascular risk for *de novo* CAD (45) and predict adverse cardiac and cerebrovascular events in patients with diabetes with or without PCI (11, 46, 47). During the follow-up period in the present study, serum 1,5-AG levels were associated with adverse clinical events, including all-cause death, CV death, and TVR. In addition to the recent report that 1,5-AG may be related to plaque rupture in diabetic patients with ACS (48), we hypothesized that the relationship between 1,5-AG and CAC in our study may be another important contributing factor supporting the hypothesis that low serum 1,5-AG levels could predict adverse outcomes. Interestingly, recent studies have reported that an intensive glucose-lowering intervention may increase serum 1,5-AG levels (49) and attenuate the progression of CAC in patients with DM (50). These findings have uncovered the possibility of early detection of CAC with serum 1,5-AG and may reflect a need for aggressive risk reduction in high-risk patients with low serum 1,5-AG levels. Further investigations regarding whether the regulation of serum 1,5-AG levels would lead to improved outcomes remain to be established.

Our study has several limitations. First, this was a single-center study with a limited sample size. Therefore, a selection bias and potential confounding factors may exist. Second, the cross-sectional nature of our study precludes cause-effect inferences on the links between 1,5-AG and atherosclerotic plaque characteristics. Third, this study was not a follow-up interventional study, and the relationship between 1,5-AG and plaque progression remains unclear. Fourth, whether the regulation of 1,5-AG would lead to improved outcomes remains to be established. A follow-up study with a larger sample size is required to determine the predictive value of 1,5-AG for plaque progression and to examine long-term outcomes after the regulation of 1,5-AG. Finally, we do not have any *in vitro* data that would help support the results of the present study.

In summary, this study revealed that low 1,5-AG levels were associated with an increased risk of CAC, as assessed by OCT, especially in DM patients. Moreover, serum 1,5-AG levels may predict future MACE in patients with DM undergoing OCT-guided PCI. These findings revealed that the measurement of serum 1,5-AG may be valuable not only in the early detection of coronary artery calcium, but also in reflecting aggressive risk reduction in high-risk patients with low 1,5-AG levels.

## Data availability statement

The raw data supporting the conclusions of this article will be made available by the authors, without undue reservation.

## Ethics statement

The studies involving human participants were reviewed and approved by the Institutional Review Board of the Taipei-Veterans General Hospital. The patients/participants provided their written informed consent to participate in this study.

## Membership of the Taipei Veterans General Hospital Coronary Intervention Study Group (TaiVeCoron)

Chuan-Tsai Tsai; Wei-Chieh Huang; Shao-Sung Huang; Tse-Min Lu (Taipei Veterans General Hospital); Chung Yu Chen (National Yang Ming Chiao Tung University Hospital); Hsin-I Teng (Cheng Hsin General Hospital); Li-Wei Chen (New Taipei City Hospital, New Taipei City Government); Hsiang-Yao Chen (Taipei Hospital, Ministry of Health and Welfare); Yen-Fu Hsu (Kinmen Hospital, Ministry of Health and Welfare); and Ying-Ying Chen (MacKay Memorial Hospital).

## Author contributions

H-IT and W-CH contributed to the conception of the study and the writing of the manuscript. H-YC, W-CH, C-TT, Y-YC, and C-HH contributed in acquiring and analyzing the data. H-IT and T-ML contributed in revising the manuscript. All authors approved the final manuscript.
